# Identification and characterization of a new species of *Taxus* — *Taxus qinlingensis* by multiple taxonomic methods

**DOI:** 10.1186/s12870-024-05338-4

**Published:** 2024-07-11

**Authors:** Xingtong Wu, Minqiu Wang, Xinyu Li, Yan Chen, Zhengping Liao, Donglin Zhang, Yafeng Wen, Sen Wang

**Affiliations:** 1 Central South University of Forestry and Technology, Changsha, Hunan China; 2 Shaanxi Academy of Forestry, Xi’an, China; 3 University of Georgia, Athens, GA USA

**Keywords:** Leaf anatomy, Lineage divergence, Niche evolution, Taxonomic status, *Taxus qinlingensis*

## Abstract

**Background:**

The taxonomy of *Taxus* Linn. remains controversial due to its continuous phenotypic variation and unstable topology, thus adversely affecting the formulation of scientific conservation strategies for this genus. Recently, a new ecotype, known as *Qinling type*, is mainly distributed in the Qinling Mountains and belongs to a monophyletic group. Here, we employed multiple methods including leaf phenotype comparison (leaf shapes and microstructure), DNA barcoding identification (ITS + *trn*L*-trn*F + *rbc*L), and niche analysis to ascertain the taxonomic status of the *Qinling type*.

**Results:**

Multiple comparisons revealed significant differences in the morphological characters (length, width, and length/width ratio) among the *Qinling type* and other *Taxus* species. Leaf anatomical analysis indicated that only the *Qinling type* and *T. cuspidata* had no papilla under the midvein or tannins in the epicuticle. Phylogenetic analysis of *Taxus* indicated that the *Qinling type* belonged to a monophyletic group. Moreover, the *Qinling type* had formed a relatively independent niche, it was mainly distributed around the Qinling Mountains, Ta-pa Mountains, and Taihang Mountains, situated at an elevation below 1500 m.

**Conclusions:**

Four characters, namely leaf curvature, margin taper, papillation on midvein, and edges were put forward as primary indexes for distinguishing *Taxus* species. The ecotype *Qingling type* represented an independent evolutionary lineage and formed a unique ecological niche. Therefore, we suggested that the *Qingling type* should be treated as a novel species and named it *Taxus qinlingensis* Y. F. Wen & X. T. Wu, sp. nov.

**Supplementary Information:**

The online version contains supplementary material available at 10.1186/s12870-024-05338-4.

## Background

Species are the basic units of biodiversity, yet distinguishing closely related species can be challenging [1]. The fundamental goals of species delimitation are not only to identify species, but also to clarify the phylogenetic relationships among and within species [2]. In addition, species delimitation also plays a vital role in the formulation of conservation policies [3–6]. Incorrect species delimitation can lead to erroneous or misleading conclusions about the evolutionary history and adaptive mechanisms of the species, consequently resulting in inappropriate conservation management decisions and adverse effects on management strategies [7, 8].

An independently evolved species (namely, the so-called good species) can be effectively delineated from different perspectives based on the classical taxonomy. However, most genealogies are always in divergence state in nature, making it challenging to discriminate species solely from one perspective [9]. More than 30 species concepts have been proposed [10], with the morphological species concept is the most commonly used [9–11]. With the development of DNA sequencing, the phylogenetic species concept has become indispensable in diagnosing species [9]. In addition, difference in the micro-structure of vegetative organs can be used as a basis for plant taxonomy [12, 13]. Furthermore, speciation is usually accompanied by the niche differentiation, and thus ecological concepts are also introduced to discriminate species [11, 14–18]. Integrating these multiple concepts enhances the rationality of species delimitation and improves the accuracy of biodiversity research results [19]. Now “the integrative species concept” is widely used in species classification [1, 9, 20].

*Taxus* is the most widespread genus with the largest number of species in Taxaceae [21, 22]. However, the classification of *Taxus* remains controversial due to its high phenotypic plasticity, niche overlapping across different lineages, and limited morphological diagnostic characters, particularly within Asian taxa [23–26]. There are three species (*Taxus wallichiana*, *Taxus fuana* and *Taxus cuspidata*) and three varieties (*Taxus wallichiana* var. *wallichiana*, *Taxus wallichiana* var. *chinensis*, *Taxus wallichiana* var. *mairei*) among the *Taxus* according to *Flora of China* (FOC) [27]. However, Farjon [22] categorized *Taxus* into five species in China (*T. wallichiana*, *T. contorta* (synonym of *T. fuana*), *T. cuspidata*, *Taxus chinensis*, and *Taxus mairei*). Möller et al. [26] divided *Taxus* into eight species (*T. cuspidata*, *T. chinensis*, *T. mairei*, *T. wallichiana*, *T*. *contorta*, *Taxus calcicola*, *Taxus phytonii*, *Taxus florinii*) and three lineages (*Qinling*, *Emei*, and *Huangshan type*) in Asia based on the morphological, cpDNA, and combined nuclear evidence (ITS and *NEEDLY*). Among these, the *Qinling type* mainly distributed from the northern parts of the Qinling Mountains to the southern parts of Taihang Mountains. *Emei type* is mostly distributed around Mount Emei and Qiaojia in the northeast of Yunnan province [28]. *Huangshan type* is mainly located in the east of China such as Huangshan, Sanqingshan, and Jiuhua Mountains [26]. Thus, *Qinling type* has a wide distribution range and obvious geographical boundary, compared with the other two lineages. *Qinling type* was thought to be closely related to *Taxus mairei* based on the *trn*L-*trn*F sequences in the phylogeographic study of *T. wallichiana* (Taxaceae) [23]. However, molecular data (*rbc*L, *mat*K, *trn*H - *psb*A, *trn*L-F) revealed *Qinling type* forming a distinct lineage and being treated as an ecotype [24, 29]. Complete plastomes analysis suggested the *Qinling type* had formed a monophyletic clade and it was closely related to *T. contorta* [25]. Möller et al. [26] conjectured that *Qinling type* was the hybrid of *T. contorta* (♀) and *T. Huangshan type* (♂) based on 13 cpDNA markers and two nuclear regions, suggesting that its morphological variation was not sufficient for species classification. Moreover, prior research has indicated phenotypic variation within the *Qinling type*, warranting further classification of its taxonomic status.

In this study, we employed “the integrative species concept” to determine the taxonomic status of the *Qinling type* based on a comprehensive analysis integrating morphological observations, microstructure of leaves, phylogenetic analysis, and niche analysis. Moreover, we established a retrieval list of *Taxus* taxonomy. Our findings aim to enhance the understanding of *Taxus* taxonomy, facilitating the formulation of effective conservation strategies for the species and improving our awareness of biodiversity.

## Results

### Comparison of morphological characters and microstructure among different *Taxus* species

**Fig. 1:**
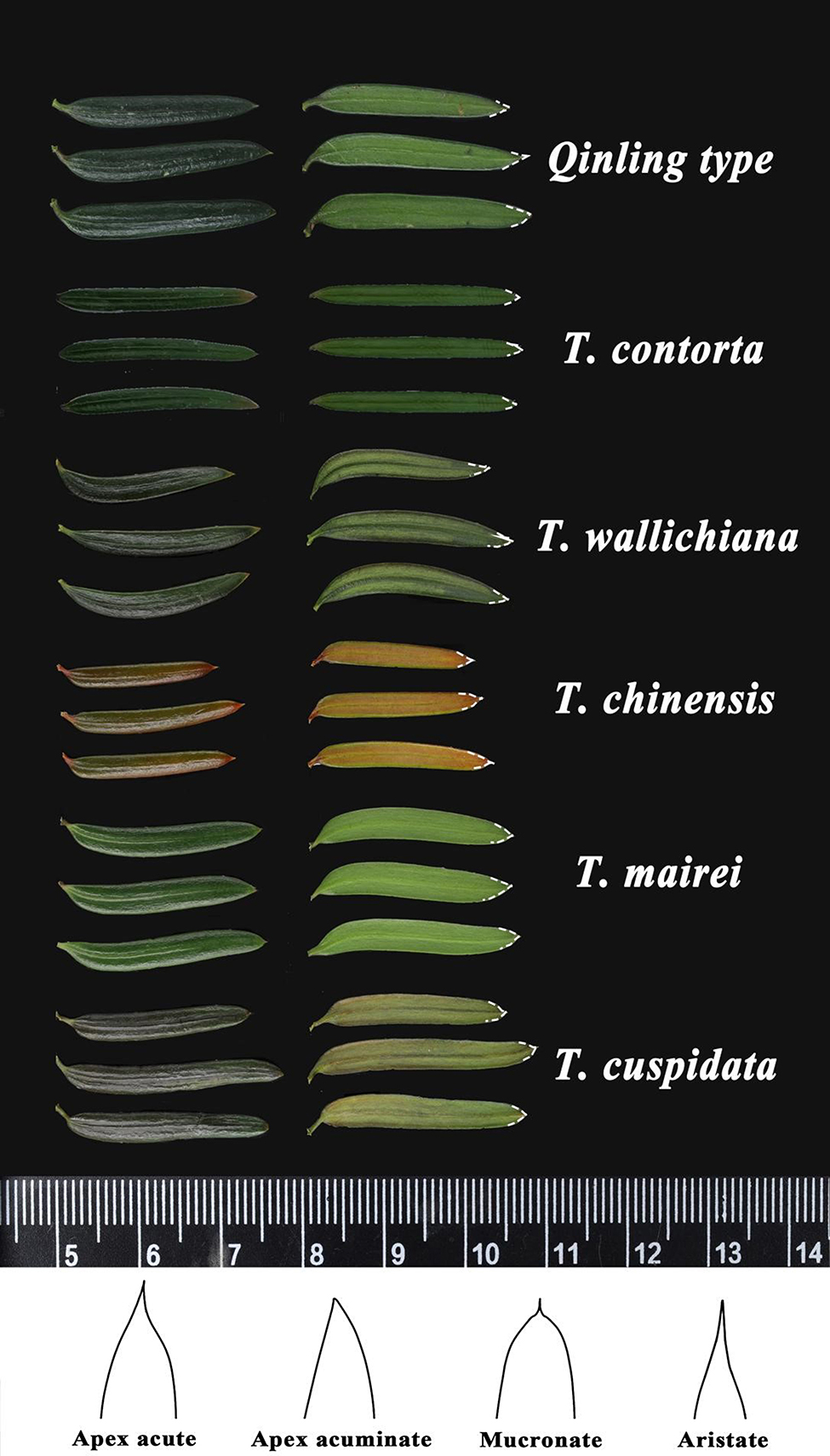
Phenotype of the six *Taxus* species (Left: the front of leaves; Right: the back of leaves) (*Qinling type* from Lushi, Henan; *T. wallichiana* from Kunming, Yunnan; *T. contorta* from Jilong, Xizang; *T. chinensis* from Jiangyou, Sichuan; *T. mairei* from Changsha, Hunan; *T. cuspidata* from Harbin, Heilongjiang)

**Fig. 2:**
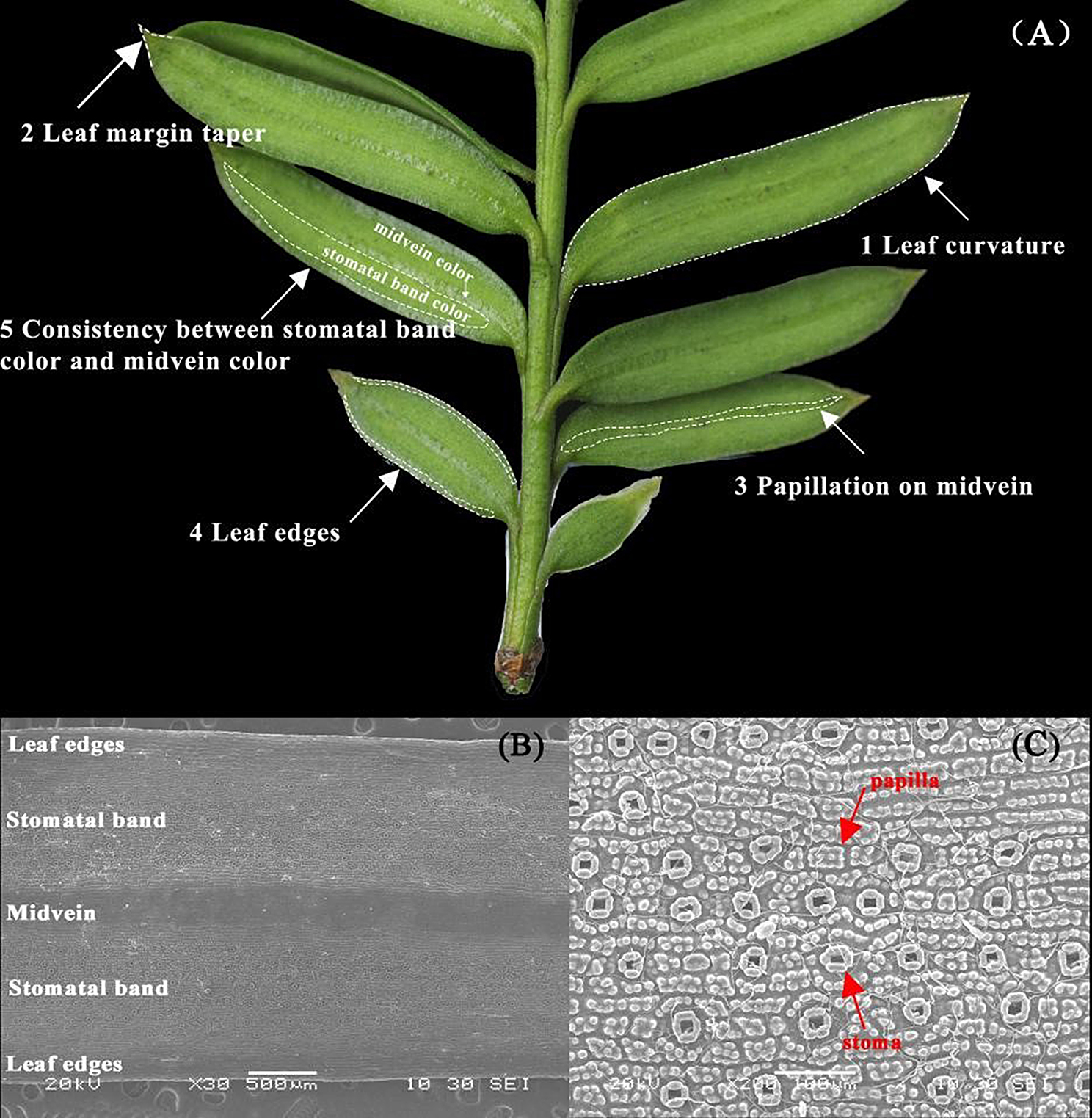
Distinguishable characteristics (**A**) for *Taxus* and scanning electron microscope (SEM) observation (**B**-**C**) to show the stomatal band, papilla, and stoma on the abaxial side of leaf

**Fig. 3:**
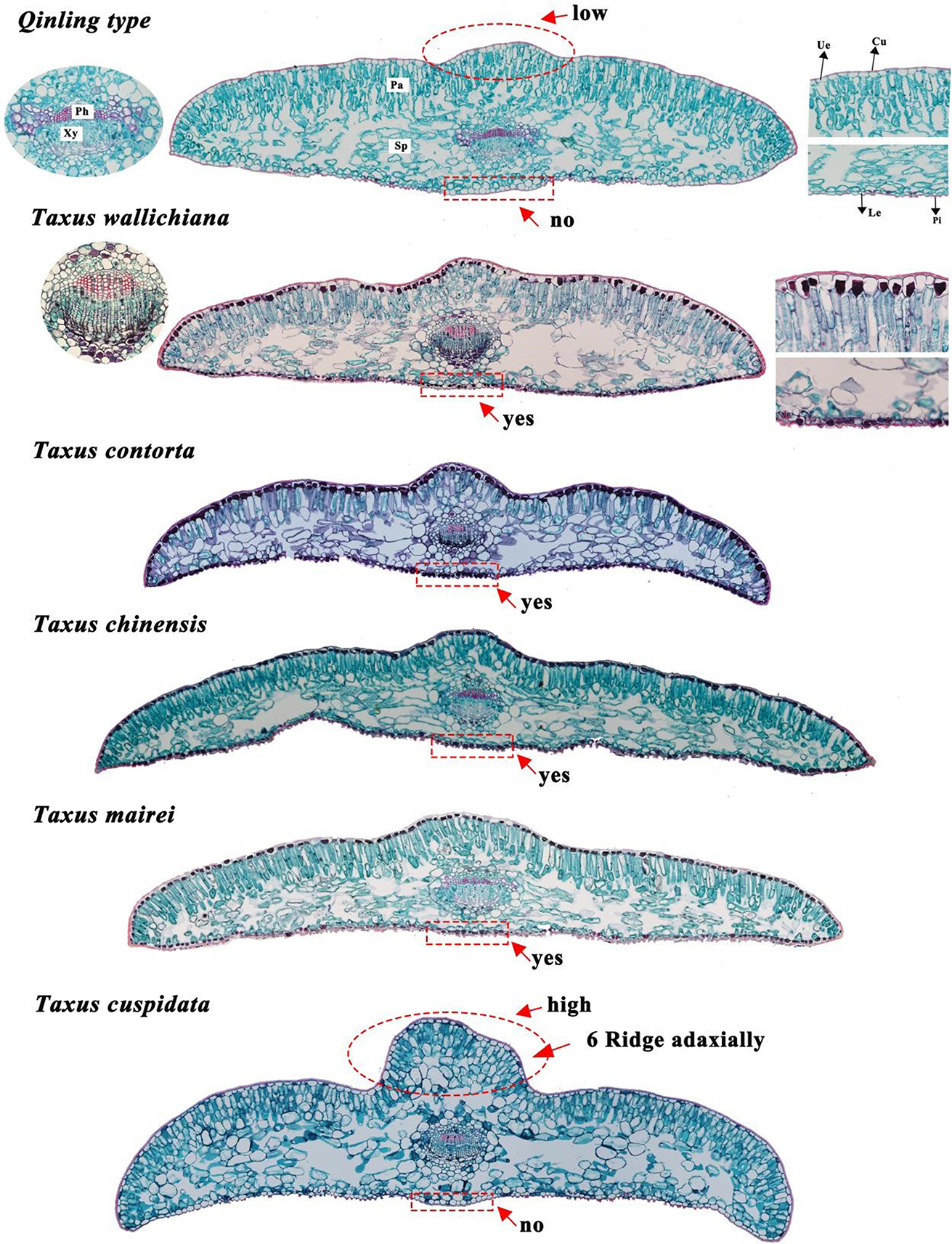
Leaf anatomy of the six *Taxus* species. *Notes* Xy: Xylem; Ph: Phloem; Pa: Palisade tissue; Sp: Spongy tissue; Cu: Cuticle; Ue: Epicuticle; Le: Lower epidermis; Pi: Papillate. Red rectangle shown whether there were cuticular papillae under the midvein; Red circle shown the rise degree of midvein on the adaxial side. The black substance shown in the epicuticle means the leaves contain tannins (Species of *T. wallichiana*, *T. contota*, *T. chinensis* and *T. mairei*)

Leaf morphology comparison of six *Taxus* species was shown in Fig. 1. It shown the differences of leaf curvature, such as falcate (*T. wallichiana*), sword-shaped (*T. contorta*), and linear (*T. chinensis*). Also, the leaf margin taper were different, particularly in tip and curvature. Leaf scanning electron microscopy (SEM) shown that stomatal apparatus and cuticular papillae are only distributed on the lower epidermis, with two stomatal bands on either side of the midvein (Fig. 2B-C). Leaf anatomy analysis indicated that *Taxus* leaves were a typical bifacial leaf, presenting the characteristic of shady plants (Fig. 3). The leaf blade is composed of three parts, epidermis, mesophyll, and veins. The epidermal is mono-layer and covered with cuticle (Cu). The mesophyll comprise palisade tissue (Pa) and spongy tissue (Sp).Within the palisade tissue, cells are irregularly shaped and arranged in single-layer, double-layer, or triple-layer, exhiting a clear hierarchal structure (Table 1). Meanwhile, spongy tissue cells are irregularly shaped, differing in cell sizes, with large gaps, and a loose arrangement (Fig. 3). Leaf veins, located centrally within the leaf, comprise xylem (Xy) and phloem (Ph). Notably, among the six *Taxus* species, only the *Qinling type* and *T. cuspidata* lack cuticular papillae under the midvein and tannins in the epicuticle (Fig. 3). However, the hard texture of leaves, the high rise of midvein on the adaxial side, and the involution of leaf edges were typical characters to distinguish *T. cuspidata* from *Qinling type*. Thus, *Taxus* species can be discriminated based on the characters of leaf curvature, margin taper, papillation on midvein, and edges.

**Table 1:** Comparison of leaf anatomical structure between *Qinling type* and other *Taxus* species

Species	Tannins	Papillate under midvein	Cell layers of palisade tissue
*Qinling type*	No	No	2–3
*T. wallichiana*	Yes	Yes	1–2
*T. contorta*	Yes	Yes	1–2
*T. chinensis*	Yes	Yes	1–2
*T. mairei*	Yes	Yes	1–2
*T. cuspidata*	No	No	2–3

**Fig. 4:**
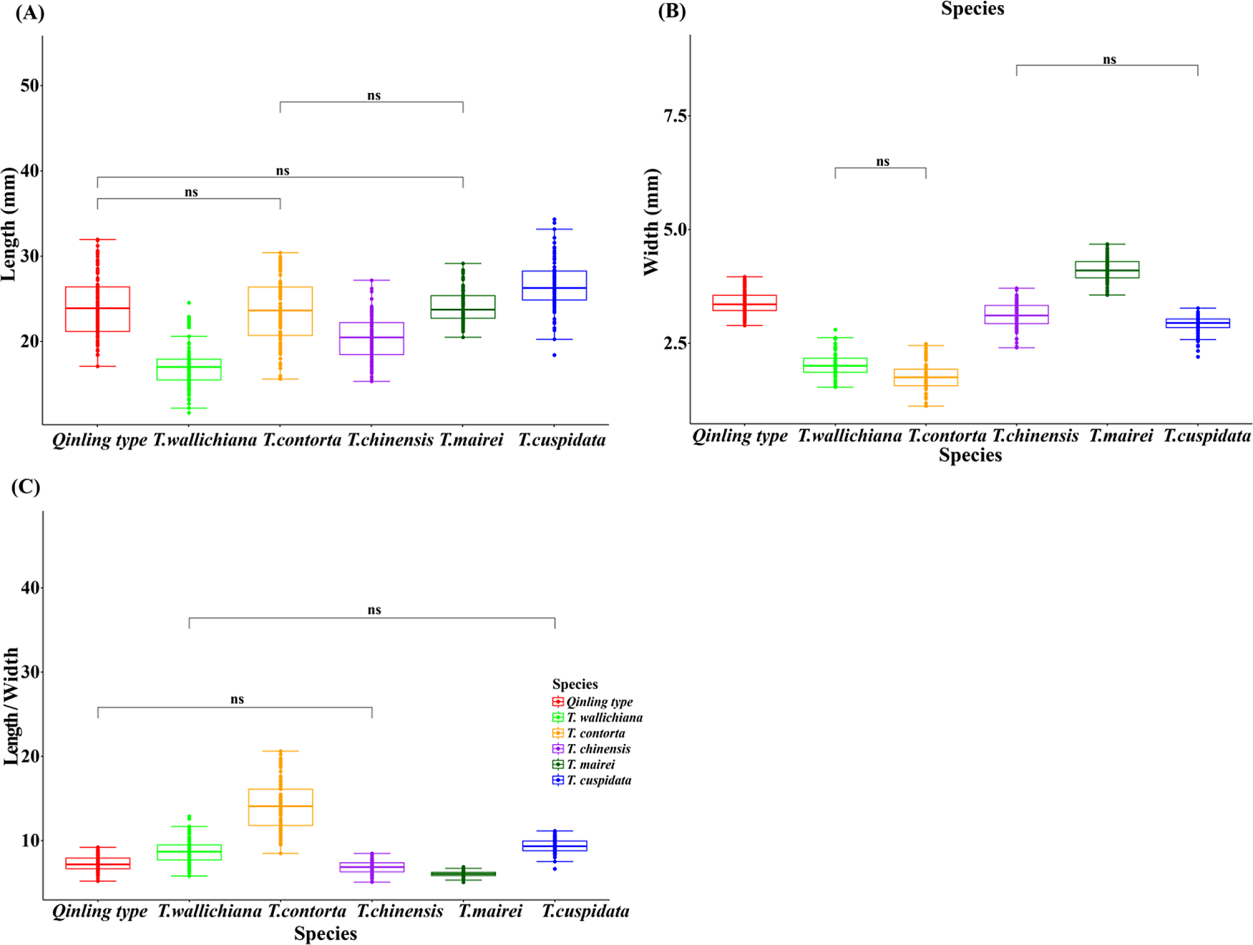
Multiple leaf comparison of the six *Taxus* species based on non-parametric test. (**A**) Leaf length, Kruskal-Wallis, χ2(5) = 308.86; (**B**) Leaf width, Kruskal-Wallis, χ2(5) = 532.62; (**C**) Leaf length/width ratio. Kruskal-Wallis, χ2(5) = 480.91. Comparisons marked “ns” did not reach significance, the other all reached significance

For leaf morphology, we compared the leaf length, width, and length/width ratio (Table S1) among the six *Taxus* species based on non-parametric statistics. Significant differences were observed in the these morphological characters in most pairwise species comparison with the following exceptions (Fig. 4, Table S1). As for the leaf length (Fig. 4A), no significant difference was found between *Qinling type* and *T. contorta*, between *Qinling type* and *T. mairei*, between *T. contorta* and *T. mairei*. Leaf width multiple comparison revealed no significant difference between *T. wallichiana* and *T. contorta*, between *T. chinensis* and *T. cuspidata* (Fig. 4B). No significant difference was observed in length/width ratio between *Qinling type* and *T. chinensis*, between *T. wallichiana* and *T. cuspidata* (Fig. 4C).

### Species delimitation

**Fig. 5:**
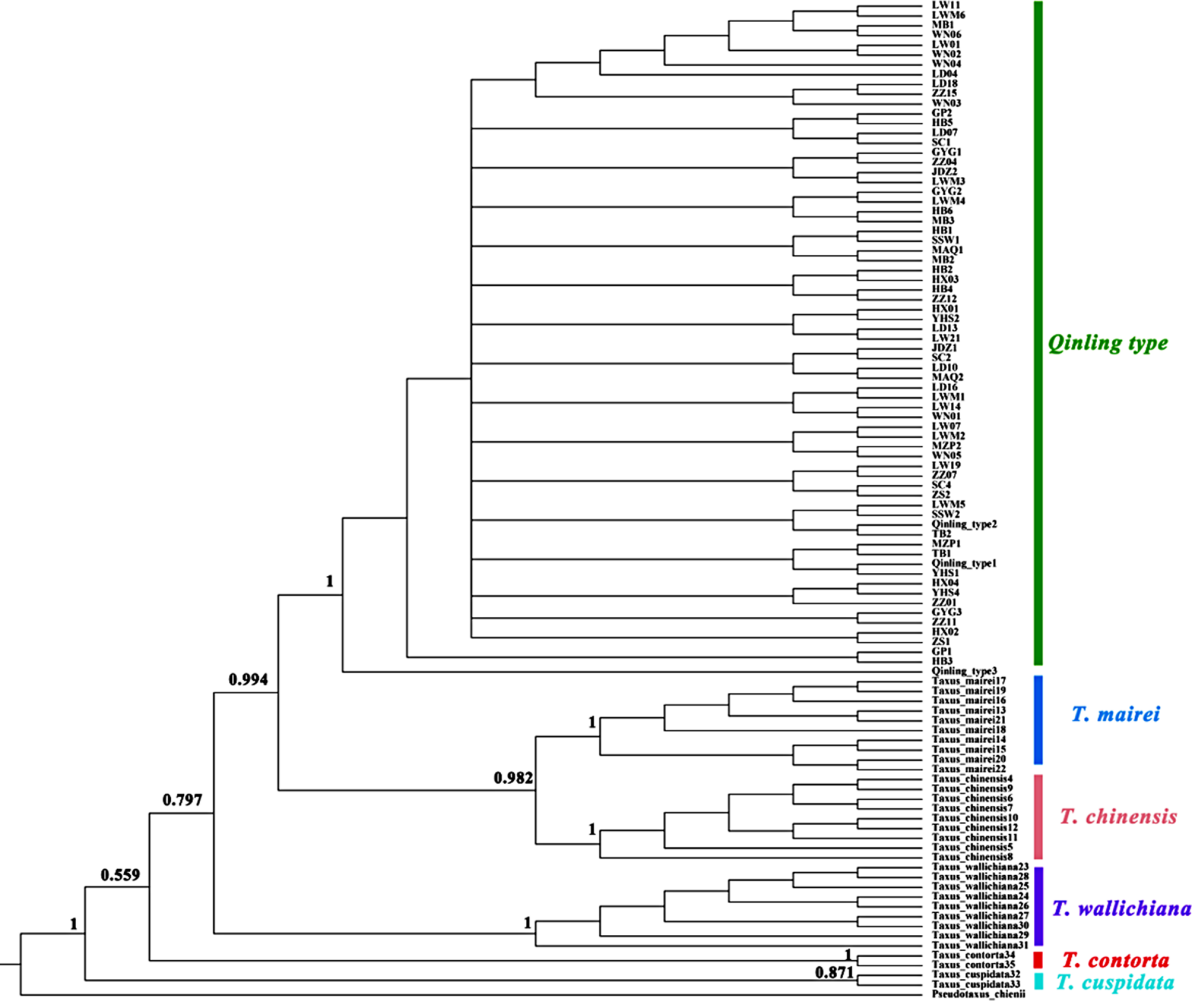
Phylogenetic tree of *Taxus* based on the DNA barcoding sequences (ITS + *trn*L*-trn*F + *rbc*L) from the Bayesian estimation

**Fig. 6:**
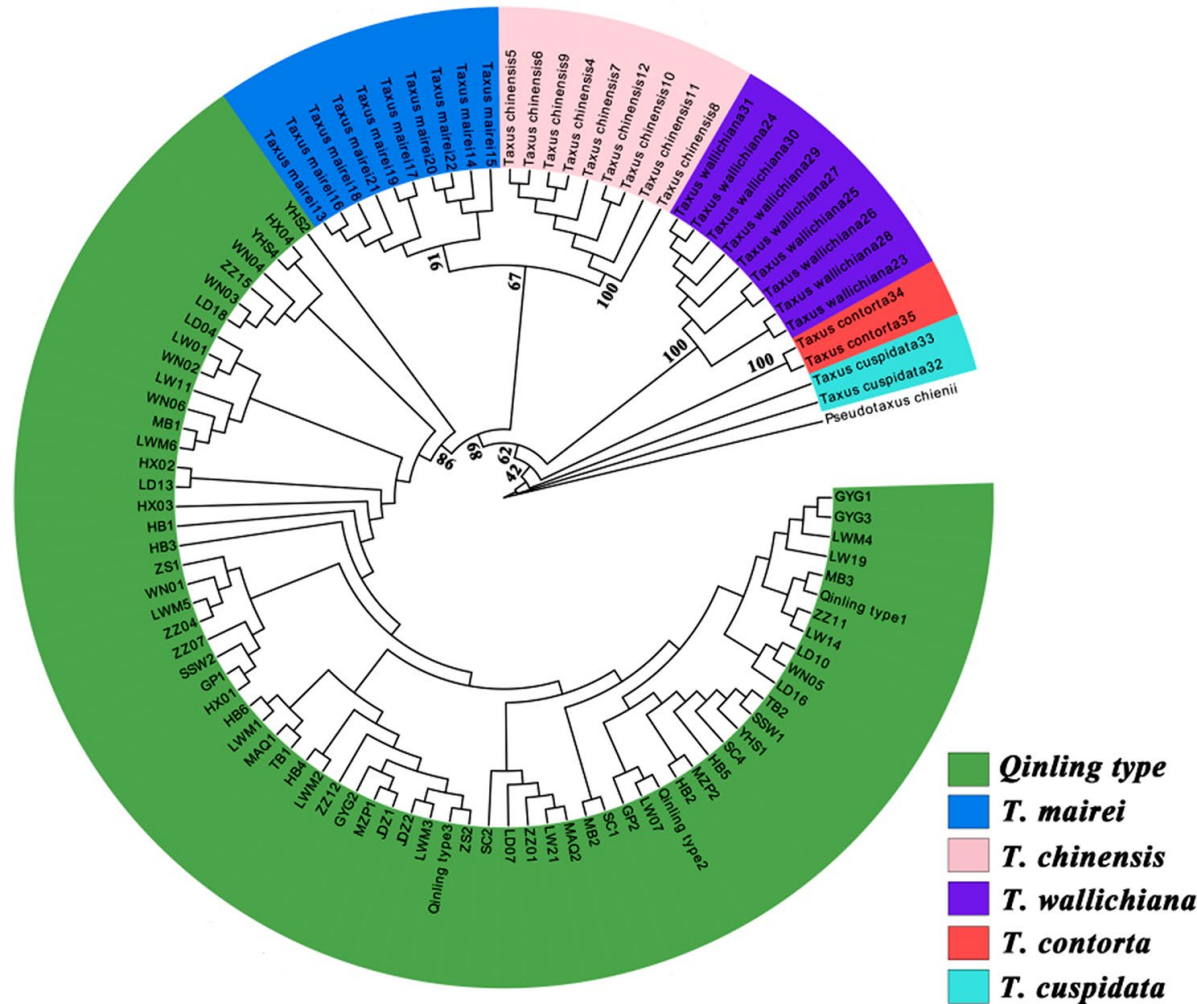
Phylogenetic tree of *Taxus* based on the DNA barcoding sequence (ITS + *trn*L*-trn*F + *rbc*L) from the Maximum likelihood estimation

The length of individual sequence for ITS, *trn*L*-trn*F, and *rbc*L, as well as the concatenated sequences (the sum of the length of the three sequences), were 1104, 947, 746, and 2797 bp, respectively. The incongruence length difference test (ILD) indicated there was no significant difference (0.05 < *p* = 1.00) in the concatenated sequence length among species. Thus, the taxonomic discrimination of *Taxus* species was conducted based on concatenated sequence. Substitution saturation test (Iss < Iss.c and *p* < 0.05) showed that the combination of the three sequences was suitable for phylogenetic analysis. Bayesian estimation (BI) and maximum likelihood estimation (ML) yielded congruent topologies (Fig. 5 and 6). 18 populations from the Qinling Mountains formed a monophyletic group and were identified as the *Qinling type*, while the other *Taxus* species also formed their respective monophyletic groups. Consequently, these results suggest that six *Taxus* species had a stable systematic position.

### Niche overlap analyses

**Fig. 7:**
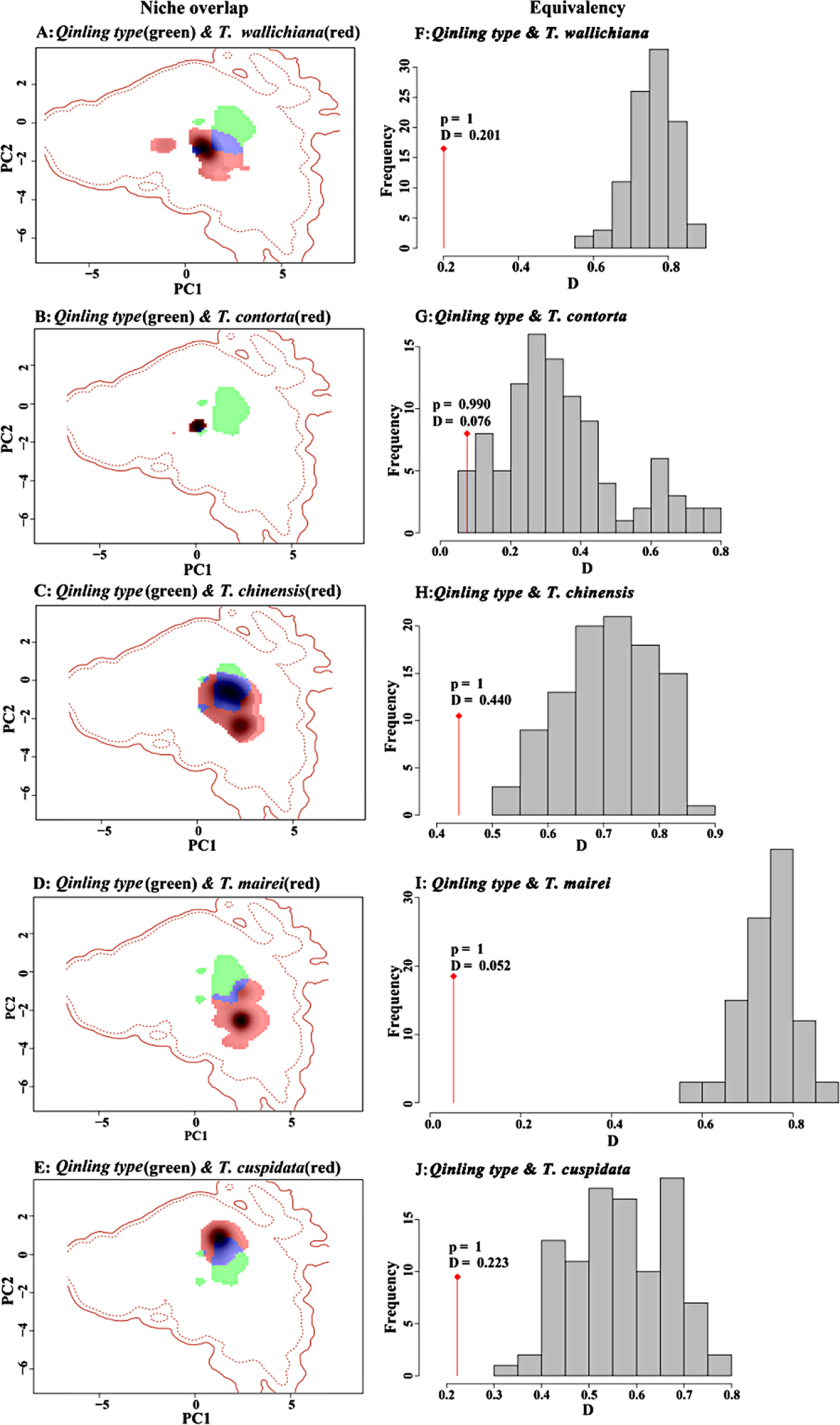
Niche quantification and overlap based on principal component analysis (PCA-env). Panel **A**-**E**: Each color (red or green) represents one species, purple represents the degree of overlap between the two species. Histograms (**F**) - (**J**) show the observed niche overlap D between the two ranges (bars with a diamond) and simulated niche overlaps (gray bars) on the tests of niche equivalency. D represents the degree of niche overlap, the significance of test is shown (Non-significant, *p* > 0.05; ***, *p* < 0.001)

Niche overlap analyses showed that *T. chinensis* had the largest niche breadth (0.310), followed by *T. cuspidata* (0.176), while the *Qinling type* (0.063) exhibited the smallest niche breadth (Fig. 7A-E). Schoener’s D metric revealed varying levels of niche overlap, ranging from very limited overlap (D = 0.052, between *Qinling type* and *T. mairei*) to moderate overlap (D = 0.440, between *T. qinlingensis* and *T. chinensis*) (Fig. 7F-J). The similarity score for ecological niche models (ENMs) constructed for the actual occurrences of the two species was lower than expected under the null hypothesis, indicating that the two species are more divergent than expected based on the available habitat for the *Qinling type* (Fig. 7F-J). However, pairwise comparisons did not yield significant difference (*p* > 0.05). Niche similarity tests showed no significant difference between the two species in pairwise comparison (*p* > 0.05), except between *Qinling type* and *T. chinensis* (*p* < 0.05) (Fig. S1 A-E).

Principal component analysis (PCA) of the driving factors (14 variables) revealed that PC1 and PC2 explained 38% and 18.5% of the total variance, respectively. Rooting conditions, elevation, and oxygen availability to roots were the dominant factors that led to the niche differentiation of the *Taxus* species in the current study (Fig. S1 F).

## Discussion

### Unique morphological characters and anatomical structure of *Qinling type*

A previous study suggested using one bud scale and 26 leaf characters to discriminate five *Taxus* species [30], discovering that *T. contorta* showed the most significant morphological difference compared to other *Taxus* species. These 27 morphological characters have been used to identify new *Taxus* species and all other species and types in Eurasia [28, 30], and lay the foundation for identifying *Taxus* species [31]. However, considering the leaf morphological characters and anatomical structure, we proposed four key morphological characters to discriminate *Taxus* species: curvature, margin taper, papillation on midvein, and edges (Fig. 2A). These characters can effectively identify *Taxus* species and simplify the species identification procedure. Additionally, significant differences were observed in multiple comparisons of leaf phenotype (length, width, and length/width ratio). Therefore, these quantitative traits can serve as indictors for categorizing *Taxus* species.

Tannins can serve as the complementary indicator for phytochemistry classification [32]. Among the six *Taxus* species, only the *Qinling type* and *T. cuspidata* lacked tannins in the epidermal cells. Therefore, the presence or absence of tannins, along with the cuticular papillae under the midvein, are two important characters to distinguish the *Qinling type* from the *T. chinensis* and *T. mairei* in their coexisting region in the warm temperate and subtropical China. Furthermore, altitude difference can also be used as one of the criterias to discriminate *Qinling type* (low altitude) from *T. chinensis* and *T. wallichiana* (high altitude) in their sympatric distribution areas, as evidenced by field investigations and prior findings [22, 33]. In conclusion, the analysis of leaf phenotype and micro-structure analysis indicated that the *Qinling type* was significantly different from other *Taxus* species, exhibiting unique morphological and anatomical characteristics.

## Stable phylogenetic position and distribution pattern of *Qinling type*

*Qinling type* was once thought to be closely associated with *T. wallichiana* var. *mairei* based on the RFLP markers [23]. Then, Liu et al. [24] proposed *Qinling type* as an independent lineage by the DNA barcoding. Fu et al. [25] pointed out that the *Qinling type* might be most closely related to *T. contorta* based on the complete plastid genomes. Möller et al. [26] proposed that the *Qinling type* is the hybrid of *T. contorta* (♀) × *T. Huangshan type* (♂) based on 13 cpDNA markers and two nuclear regions, and it was an independent lineage. In current study, *Qinling type* formed a monophyletic lineage based on one nuclear regions (ITS) and two cpDNA makers (*trn*L*-trn*F + *rbc*L), and its divergence time was prior to *T. chinensis* and *T. mairei* (Fig. 5 and 6). Taken together, these results confirmed that *Qinling type* is an independent lineage. However, the position of *Qinling type* in the phylogenetic tree is constantly changing. The inconsistent findings of *Qinling type* may be attributed to the conflicts between nuclear genomes and chloroplast genomes, the ancient hybridization, and the reciprocal chloroplast capture [34, 35]. From the phylogenetic perspective, the *Qinling type* represent a steady lineage. This may be attributed to geographic isolation and short migration distances, leading to reduced gene exchange between different species over time [36–38].

Previous studies have revealed that *Rhinopithecus brelichi* [39] and *Juglans regia* [40] all originated from ancient hybridization events, with their respective hybridization periods being 1.8 Mya and 3.45 Mya. In *Taxus* species, Qin et al. revealed the divergence between *T. wallichiana* and *T. florinii* occurred in 5.9 Mya (± 0.39) based on population-genomic analysis from dd-RAD data [41]. This time approximately aligns with the predication made by Möller et al. [26] (4.9–6.8 Mya). Furthermore, Möller et al. [26] also proposed that the hybridization event of *Qinling type* occurred in the late Miocene, around 6.8 Mya (95% HPD = 1.9–12.7 Mya) years ago. Hence, *Qinling type* belongs to the ancient hybridization event rather than a recent one.

Möller et al. [28] investigated the geographical distribution of the *Qinling type* and found it mainly distributed around the western Sichuan basin, spanning from the northern Qinling Mountains to the southern Taihang Mountains, indicating a relatively independent niche of the *Qinling type* in terms of geographical distribution and elevation. Furthermore, our previous field investigation showed that *Qinling type* predominantly inhabited the lower elevations of the Qinling Mountains and Taihang Mountains. When *Qinling type* was sympatric with *T. chinensis* and *T. wallichiana*, its altitude was lower than 1500 m [28, 42]. Nevertheless, our niche similarity tests demonstrated that *Qinling type* had niche overlap with *T. chinensis*, possibly because niche overlap analysis fails to capture the vertical spatial niche differences between the two species [16]. Notably, elevation was the main driving factor in the differentiation of *Taxus* species from the PCA-env analysis and species distribution models (SDMs) [33].

### Suggestions for future research priorities on *Taxus*

DNA barcoding is a powerful taxonomic tool to identify and discover species [43–45]. Liu et al. [29] showed that *trn*L - *trn*F is an ideal barcode for *Taxus*, effectively distinguishing all *Taxus* species and attaching to ITS for hybird identification. The purpose of this study was to determine the taxonomic status of the *Qinling type.* Therefore, we used the combination of ITS + *trn*L*-trn*F + *rbc*L to identify *Taxus* species. Möller et al. [26] reconstructed the phylogeny of the extant *Taxus* lineages, yet only one sample was included in each taxon. In our study, a total of 66 individuals of *Qinling type* were collected from 18 populations in the Qinling mountains to confirm its taxonomic status. We found *Qinling type* showed obvious phenotypic and niche differenation compared with the other *Taxus* species, and it formed an independent lineage. Furthermore, we have completed Double Digest Restriction Associated DNA (dd-RAD) Sequencing for *Qinling type* and its related species in their sympatric distribution. Phylogenetic analysis, admixture analysis, and demographic analysis indicated that *Qinling type* was an independent lineage not originated from recent hybridization (unpublished data). In addition, *Qinling type* is also found in Sichuan and Yunnan provinces [29]. However, due to the sporadic distribution of these samples, they were not included in this study (morphological and phylogenetic analysis). Next, we will further to explore the origin and diffusion corridors of *Taxus qinlingensis* based on a more comprehensive sampling strategy.

According to the International Union for Conservation of Nature (IUCN), three *Taxus* species (*T. wallichiana*, *T. contorta*, and *T. cuspidata*) are classified as endangered, while two *Taxus* species are considered vulnerable (*T. chinensis* and *T. mairei*) (IUCN, 2022a) [46]. In China, all the *Taxus* species are under the first-grade state protection (National Forestry and Grassland Administration, 2021). In general, *Taxus* species are dioecious [22]. However, our previous investigation found that some individuals of *T. qinlingensis* around the Qinling Mountains were monoecious (unpublished data). Up to now, the sex transformation mechanisms of *Taxus* remain largely unknown [47, 48]. Generally speaking, the mechanisms of sex determination systems including genetic sex determination (GSD), environment sex determination (ESD), and the combination of GSD and ESD [49]. These mechanisms reveals that plants have to adopt different strategies to cope with diverse biotic and abiotic stresses for survival in adverse environmental conditions [50, 51]. In the future, with the application of whole-genome sequencing [52–54], the conservation strategies for *T. qinlingensis* can be explored from its habitat water scarcity, forest regeneration mechanism, seed after-ripening, and sex determination mechanism [55, 56].

## Conclusion

This study employed a combination of leaf phenotype comparison, phylogenetic analysis, and niche analysis to reveal the classification status of the *Qinling type*, and we named it *Taxus qinlingensis* Y. F. Wen & X. T. Wu sp. nov (with the detailed information provided in Taxonomic Key). Soil conditions and elevation played a vital role in the speciation of *Taxus*. These results enhance the importance of using “the integrative species concept” in species categorization, and reasonable species assessments will help to raise awareness of the importance of biodiversity. Future studies should focus on the origin and diffusion path of *Taxus qinlingensis*, exploring its sex determination locus.

## Methods

### Sample collection and DNA extraction

**Fig. 8:**
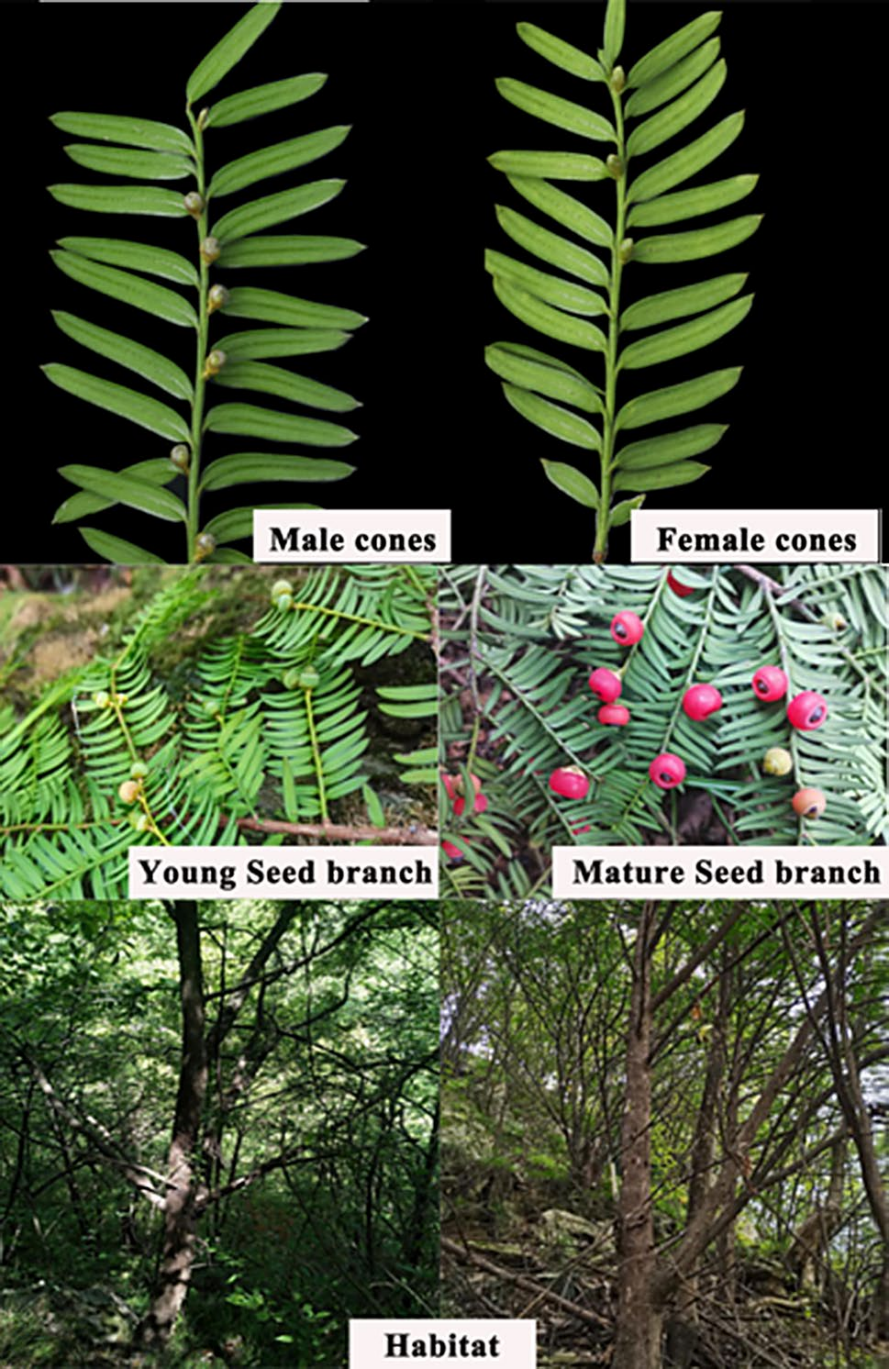
Habitat, seed branch and cones of *Qinling type*

Several field investigations of *Qinling type* were performed from the year 2019 to 2022, covering the northern part of Qinling Mountains to the southern part of Taihang Mountains. Habitat, seed branches, and cones of *Qinling type* were shown in Fig. 8. A total of 66 samples were collected from 18 *Qinling type* populations, with 2–6 samples per population. Detailed information of species and populations were presented in Table S2. The formal identification of all voucher specimens was identified by Professor Yafeng Wen according to Flora of China (1999) [27] and previous studies [42], and subsequently deposited in the herbarium of Central South University of Forestry and Technology. Accession numbers of the specimens are shown in Table S3. DNA was extracted from fresh samples by a modified 2×CTAB (Cetyltrimethylammonium Bromide) protocol [42]. The quality and integrity of DNA were evaluated by 1.0% agarose gels.

### Morphological characters comparison among different species

Considering the wide applicability and popularity of Farjon’s classification system [22], we gathered five *Taxus* species to analyze their morphological characteristics, namely *T. wallichiana*, *T. contorta*, *T. cuspidata*, *T. chinensis* and *T. mairei*. Among them, *T. contorta* and *T. mairei* were most closely related to *Qinling type*, while *T. chinensis* and *Qinling type* are sympatrically distributed across the Qinling mountains [23, 25, 26]. These *Taxus* species were gathered from June to August in 2022, and their distribution is shown in Fig. S2. Six characters were employed, with their respective character states were defined in Fig. 2 and 3, and Table 2. For each species, ten mature leaves were selected for measuring leaf length (LL) and leaf width (LW, leaf blade at widest point). Ten measurements were taken for each mature leaf, resulting in a total of 100 measurements for each species. Length/width (L/W) is the ratio of leaf length (LL) to leaf width (LW). Considering the data did not conform to the normal distribution and homogeneity test of variance, significant differences among different *Taxus* species were analyzed using Kruskal-Wallis’s multiple range tests based on non-parametric statistics in “ggpubr” and “rstatix” package [57, 58]. Statistically significant differences between two *Taxus* species (*p* ≤ 5.0e-3) were indicated as “*” .

**Table 2:** Qualitative characters used in the morphological comparison of *Taxus* (reference to [30])

Character number	State	Type	State	Species
1	Curvature	Discrete	Linear	*T. chinensis*
			Falcate	*T. mairei*, *T. wallichiana*
			Sword-shape	*T. contorta*
			Slightly falcate	*Qinling type*, *T.cuspidata*
2	Margin taper	Discrete	Shown in Fig. 1	
3	Papillation on midvein	Discrete	Absent	*Qinling type*, *T.cuspidata*
			Scattered	*T.wallichiana*
			Dense	*T. chinensis*, *T. contorta* , *T. mairei*
4	Edges	Discrete	Flat	*Qinling type*, *T.wallichiana*, *T. chinensis, T.mairei*
			involution	*T. contorta, T. cuspidata*
5	Consistency between stomatal band color and midvein color	Discrete	Same	*T. chinensis*, *T. contorta*
			Different	*Qinling type*, *T.mairei*, *T. cuspidata*, *T.wallichiana* ,
6	Ridge adaxially	Discrete	High	*T. cuspidata*
			Median	T. *mairei*, *T. wallichiana* , *T. contorta*, *T. chinensis*
			Low	*Qinling type*

### Microstructure observation of the leaf and lower epidermis

To observe the differences in leaf microstructure among the *Taxus* species, healthy and mature leaves were collected from the middle part of the tree and washed with distilled water. Square leaf samples with 3 mm×3 mm size were cut off at 1/3 distance from the base of the leaf, fixed by FAA fixative (containing 37% formaldehyde, glacial acetic acid, 70% ethyl alcohol; 1:1:18), labeled, and stored at 4℃ in the refrigerator. By the conventional paraffin sectioning method [59], the slices with a thickness of 10 μm were prepared as follows: leaf samples were dehydrated, transparentized, wax dipped, embedded, sectioned, dewaxed, rehydrated, stained (safranin O - fast green), dehydrated, transparentized (dimethylbenzene), and sealed (neutral balsam). Photographs of the sections were taken by Leica inverted microscope equipped with a 10× and 20× objective lens, respectively.

Scanning electron microscope (SEM) observation was used to make the stomatal band and papilla on the abaxial side more clearly. First, leaves were put into the stationary liquid of glutaric dialdehyde (2.5%). Then, the leaves were washed with PBS buffer (biosharp) and distilled water, dehydrated in different concentrations of alcohol (30%, 50%, 70%, 80%, 90%, 95%, 100%), transitioned to tert-butanol, and then freeze-dried. After drying, the samples were plated in a GVC-1000 ion sputterer for 20 min and then subjected to scanning electron microscopy (SEM) on a JSM-6380LV for sub-microscopic observation. Representative fields of view were selected to observe the leaf morphology and photographed for analysis.

### Barcoding sequencing and sequence assembly

One nuclear internal transcribed spacer sequence (ITS) and two chloroplast DNA fragments (*trn*L*-trn*F and *rbc*L) were chosen as DNA barcoding to identify *Taxus* species by the results reported by Liu et al. [29](Table S4, [60–62]). PCR amplification was carried out in 20 µL reaction system containing 15 µL 2 × Taq PCR Master mix, 1 µL forward primer, 1 µL reverse primer, and 3 µL DNA template. PCR amplification procedures for each sequence are shown in Table S3. PCR products were inspected on 1.0% agarose gels, and purified using the SanPrep Column PCR Product Purification Kit, and the specific DNA fragments were recovered using the SanPrep Column DNA Gel Extraction Kit. Subsequently, the purified PCR products were sequenced in both directions on ABI3770XL DNA Sequencer (Applied Biosystems, Foster City, CA, USA). Furthermore, one sequence of *Pseudotaxus chienii*, three sequences of *Qinling type*, and thirty-two sequences of *Taxus* were downloaded from NCBI for phylogenetic analysis (Table S3). Consequently, this study incorporated *Qinling type* samples from the provinces of Shanxi, Henan, and Shaanxi.

Firstly, nucleotide sequences were aligned using MAFFT v7.471 with default parameters [63]. Secondly, the sequences were manually adjusted using MEGA X [64]. Thirdly, sequence fragments were made into the combined matrix by phylotools package (https://github.com/helixcn/phylotools). Afterwards, the incongruence length difference test (ILD) was carried out using PAUP 4.0 to determine whether the supermatrix was suitable for subsequent analyses [65, 66]. Finally, the saturation of the supermatrix was determined through substitution saturation test using DAMBE [67].

### Phylogenetic relationship

The phylogenetic relationship was assessed based on the combination of the three sequences (ITS + *trn*L-*trn*F + *rbc*L). Bayesian inference (BI) analyses were conducted by Mrbayes 3.2.6 [68] with the GTRGAMMA nucleotide substitution model determined by PartitionFinder2 [69] in PhyloSuite software [70] according to the principle of minimum Akaike information criterion (AIC). Then, the maximum likelihood (ML) analyses were conducted using GTRGAMMA model in RAxML v 8.2.X with 1000 bootstrapping replicates [71]. *Pseudotaxus chienii* were rooted as the out-group for all the analysis. The cladogram tree was edited by the Evolview software [72].

### Niche equivalency and similarity tests

19 bioclimatic variables and elevation were downloaded from WorldClim 2.1 (https://worldclim.org) with a spatial resolution of 2.5 min from 1970 to 2000. To avoid biased estimates of model coefficients and spurious significance levels resulting from multicollinearity, we excluded highly correlated climate variables based on Pearson’s correlation coefficient (|r|>0.75), finally, six climatic variables (Bio2, Bio3, Bio11, Bio15, Bio18 and elevation) were retained. Slope and aspect were obtained from Digital Elevation Model using the 3D analyst tools in the software ArcGIS 10.4. In addition, five soil variables were downloaded from the Harmonized World Soil Database (HWSD,https://www.fao.org/soils-portal/data-hub/soil-maps-anddatabases/harmonized-world-soil-database-v12/en/) and one human interference index (HII) from Socioeconomic Data and Applications Center (SEDAC, http://sedac.ciesin.columbia.edu) based on previous studies [33, 73, 74]. Finally, a total of 14 variables were retained for subsequent analysis (Fig. S3).

Ecological niche models and multivariate analysis in the “ecospat” package were used to quantify and compare the niches in geographic space and environmental space between two species (including subspecies and varieties) [16, 75, 76]. The degree of niche overlap between two species was estimated using the Schoener’s D index, ranging from 0 (no niche overlap) to 1 (perfect niche overlap) [16, 77]. Rödder et al. [78] proposed the following grading: 0-0.2 = no or very limited overlap, 0.2–0.4 = low overlap, 0.4–0.6 = moderate overlap, 0.6–0.8 = high overlap, 0.8-1.0 = very high overlap. Niche equivalency test evaluated the congruence of the two species’ geographic niches, whereas the niche similarity test was conducted to investigate the similarity between actual environmental niches and the expected ones [16]. The null hypothesis of niche equivalency is rejected when empirical values are significantly less than the critical values, indicating the two niches are not equivalent to each other [19, 79]. Hypotheses of niche similarity (i.e., the hypothesis of niche conservatism) are accepted when there is significant difference (*P* < 0.05) between the empirically observed Schoener’s D values (i.e., niche overlap) and the expected simulated overlap from the 100 pseudo-replicated datasets [18]. Furthermore, the contribution rate of driving factors to niche differentiation was calculated by the “factoextra” package [80].

### Taxonomic treatment

#### Taxus qinlingensis

Y. F. Wen & X. T. Wu sp. nov. (秦岭红豆杉 is the Chinese name of ***Taxus qinlingensis***.)

#### Diagnosis

This newly species resembles *Taxus mairei* in possessing similar leaf phenotype and elevation distribution, yet it is distinct due to the absence of papillate on midvein and tannis in the epidermis.

#### Type

CHINA. Shanxi Province: Xi’an City, Zhouzhi County, 108.23 N, 33.87 E, elev. 1100 m, July 23, 2019. Yafeng Wen WZZ201901 (holotype: CSFI).

#### Description

Evergreen tree to 25 m, trunk to 1.3 m DBH (diameter at breast height). Diaecious or monoecious. Flowering from February to April, fruiting from September to December. Bark thin, light red, purple-brown, or gray, split into strips or irregular flakes. Branches are numerous, ascending to erect, then spreading or drooping, forming a spreading, rounded, or pyramidal crown. Foliage branchlets irregularly alternate, terete with fine grooves alongside decurrent leaf bases, yellowish green turning green to brown. Winter buds are ovoid, scales persistent at base of shoots. Leaves distichously arranged, relatively loose on branchlets with leaf base twisted and spirally inserted, nearly sessile, thinned leathery, linear or falcate, 2.0–3.0 cm long, 2.5–3.5 mm wide, apex acute and aristulate, with revolute margins. Leaf adaxial side color was yellowish green to dark green with glossiness. Leaf abaxial side has two yellowish stomatal bands with an irregular arrangement, no papillae on the midvein, and both the midvein and the leaf edges color are shiny. Midvein length was 300–400 μm. Male cones axillary, solitary, ovoid with bracts at the base, with short stems under cones, and form rows on either side along the fertile shoots, yellowish green to yellow, 8–14 peltate microsporophylls, each with 4–6(-8) pollensacs. Female cones axillary, solitary, ovoid, and subsessile. Aril green at first, covering the lower part of the seed, swelling to succulent red (usually more translucent), and cover seeds, leaving its apex free, cup-like, 8–10 mm long, 7–10 mm wide. Seeds ovoid or obovoid, slightly flattened, with two obtuse ridges and a protruding apex, 5.0–8.0 mm long, 3.5–5.0 mm in diameter, green to brown or black.

#### Distribution and habitat

*Taxus qinlingensis* is mainly grown in deciduous broad-leaved forests on mountain slopes at altitudes between 500 and 1600 m in Shaanxi, Henan, Shanxi, and Hubei provinces. However, it can also found in the theropencedrymion of Yunnan and northwestern Sichuan (Danba, Songpan), where elevations ranging from 1700–2000 m and 2300–2500 m, respectively.

#### Specimens examined

CHINA. **Shaanxi province**: Zhouzhi county, evergreen mixed angiosperm/deciduous broad-leaved forest, 990–1150 m, 23 July 2019, M. Q. Wang & Y. F. Wen (Herbarium, Central South University of Forestry and Technology, CSFI); Weinan city, mixed angiosperm/conifer forest hill, 1450–1500 m, 1 August 2019, M. Q. Wang & X. T. Wu (CSFI); Zhashui county, mixed angiosperm/conifer forest hill, 900–1000 m, 18 April 2021, Z. P. Liao (CSFI); Foping county, mixed angiosperm/conifer forest hill, 900–1000 m, 3 March 2022, Z. P. Liao (CSFI); Lantian county, mixed angiosperm/conifer forest hill, 1000–1200 m, 31 March 2022, Z. P. Liao (CSFI). **Gansu province**: Liangdang county, mixed angiosperm/conifer forest on hill, 1100–1200 m, 30 July 2019, M. Q. Wang & Y. F. Wen (CSFI). **Henan province**: Lushi county, evergreen mixed angiosperm/deciduous broad-leaved forest, 1000–1300 m, 12 December 2022, X. T. Wu (CSFI).

#### Conservation status

Endangered (EN A2cd; C1). The entire area of occupancy (AOO) of this species in nature is around 164 km^2^. Some are distributed around the village, known as the fengshui trees, while others are distributed around the mountains that are not easy to access. The total number is less than 30,000. *Taxus* exhibits weak regeneration ability and poor habitat quality. Consequently, we proposed that *T. qinlingensis* should be classified as Endangered (EN) by IUCN standards [81–83].

### Key to the *Taxus* species

**Table:** 

1 midvein without papillae	2
1 midvein with densely and evenly distributed papillae	3
2 Leaves thinned leathery, linear, 2.0–3.0 cm × 2.5–3.5 mm, leaf margins parallel, apex acute, midvein color different from stomatal band color, leaf edges shiny on abaxial leaf surface, with relatively low leaf length/width ratio (6.50–8.06, mean > 7).	*T. qinlingensis*
2 Leaves leathery, linear and straight, 2.0–4.0 cm × 2.0–3.5 mm, leaf edges involution, midvein convex, apex mucronate, midvein color different from stomatal band color, leaf edges shiny on abaxial leaf surface, with relatively high leaf length/width ratio (9.00-10.25, mean > 9).	*T. cuspidata*
3 Leaves leathery, linear, 1.5–2.5 cm × 2.5–3.5 mm, leaf margins parallel, apex acute, midvein color same as stomatal band color, leaf edges not shiny on abaxial leaf surface, with relatively low leaf length/width ratio (5.87–7.20, mean > 6)	*T. chinensis*
3 Leaves texture relatively hard, falcate, 1.0–2.5 cm × 1.5–3.0 mm, leaf margins parallel, apex acuminate, midvein color different from stomatal band color, leaf edges shiny on abaxial leaf surface, with relatively high leaf length/width ratio (5.00–13.00, mean > 8)	*T. wallichiana*
3 Leaves thinned leathery, sword-shaped, 1.2–2.5 cm × 1.0–3.5 mm, leaf margins parallel, apex acute, aristate, midvein color same as stomatal band color, leaf edges not shiny on abaxial leaf surface, with relatively high leaf length/width ratio (8.00–25.00, mean > 10)	*T. contorta*
3 Leaves thinned leathery, slightly falcate, 2.0–3.5 cm × 2.5–4.0 mm, leaf margins parallel, apex acuminate, midvein color different from stomatal band color, leaf edges shiny on abaxial leaf surface, with relatively low leaf length/width ratio (5.87–7.15, mean > 6)	*T. mairei*

### Electronic supplementary material

Below is the link to the electronic supplementary material.

Supplementary Material 1

Supplementary Material 2

## Data Availability

The original phenotypic datasets in current study are available in Table S5. All sequences (ITS, *trn*L - *trn*F and *rbc*L sequences) used in this study have been submitted to the National Center for Biotechnology Information (NCBI, https://www.ncbi.nlm.nih.gov/) with accession numbers (MW788517 - MW788520, MZ208838 - MZ208861, and OQ891381 - OQ891399 for ITS sequence; MW792129 - MW792132, MZ220779 - MZ220802, and OQ978853 - OQ978871 for *trn*L - *trn*F sequences; MW792092 - MW792095, MW893342 - MW893365, and OQ913085 - OQ913103 for *rbc*L sequence) (Table S3). All the sequences have been available online. Information for other samples used for phylogenetic analysis download from NCBI can be found in Table S3.
