# Supplementary material for: Identification and characterization of a new species of Taxus — Taxus qinlingensis by multiple taxonomic methods
Source: BMC Plant Biol. 2024 Jul 11;24:658. doi: 10.1186/s12870-024-05338-4 (PMC11238484; doi:10.1186/s12870-024-05338-4)
Supplement: Supplementary file 2 — Supplementary Material 2 [file 12870_2024_5338_MOESM2_ESM.docx]

**Supplementary file**


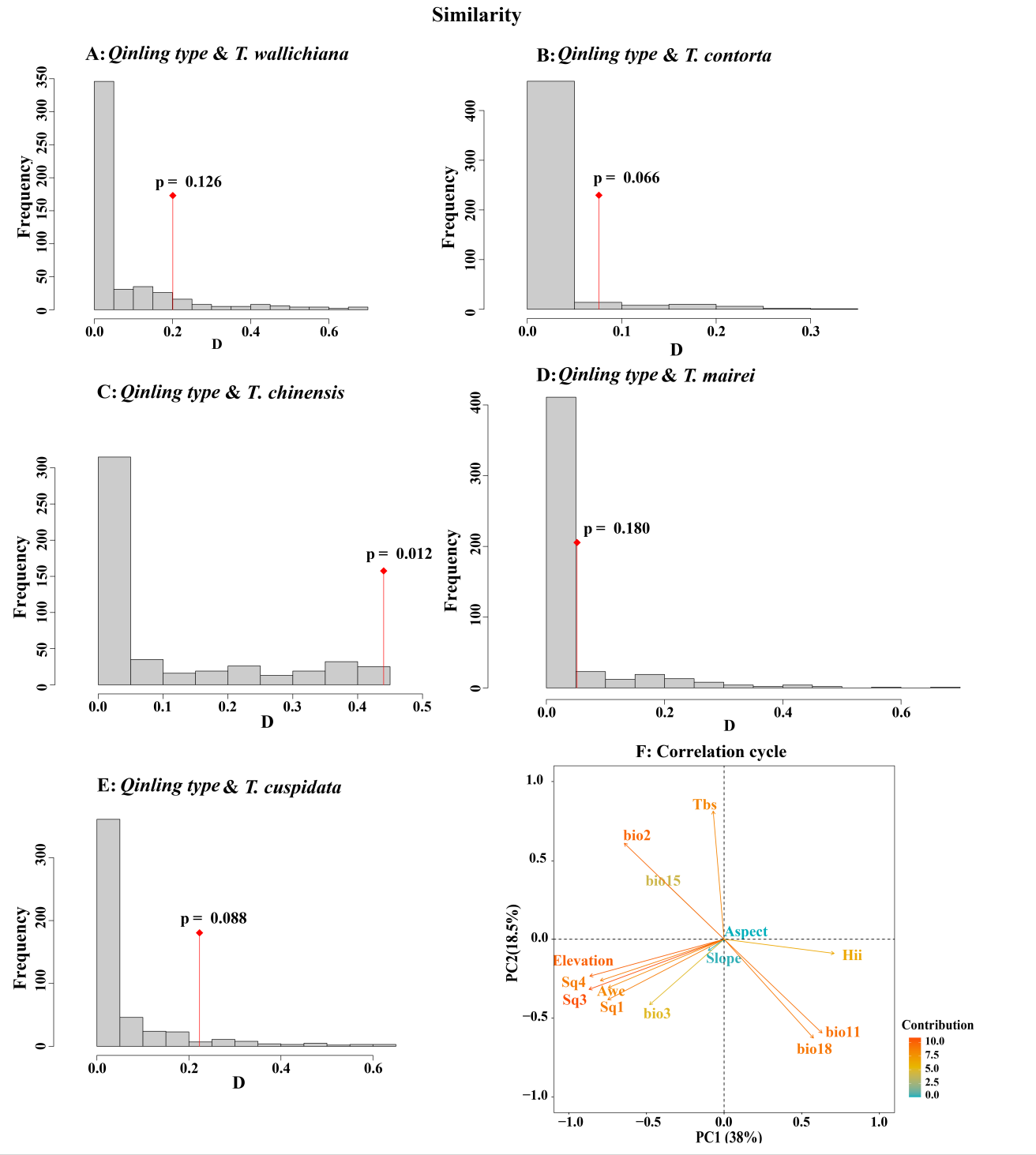


**Fig. S1** Similarity test between the *Qinling type* and other *Taxus* species. Notes: The meaning of variables are the same as Figure S2. p > 0.05 represents there is no significant differences between the two species.

**
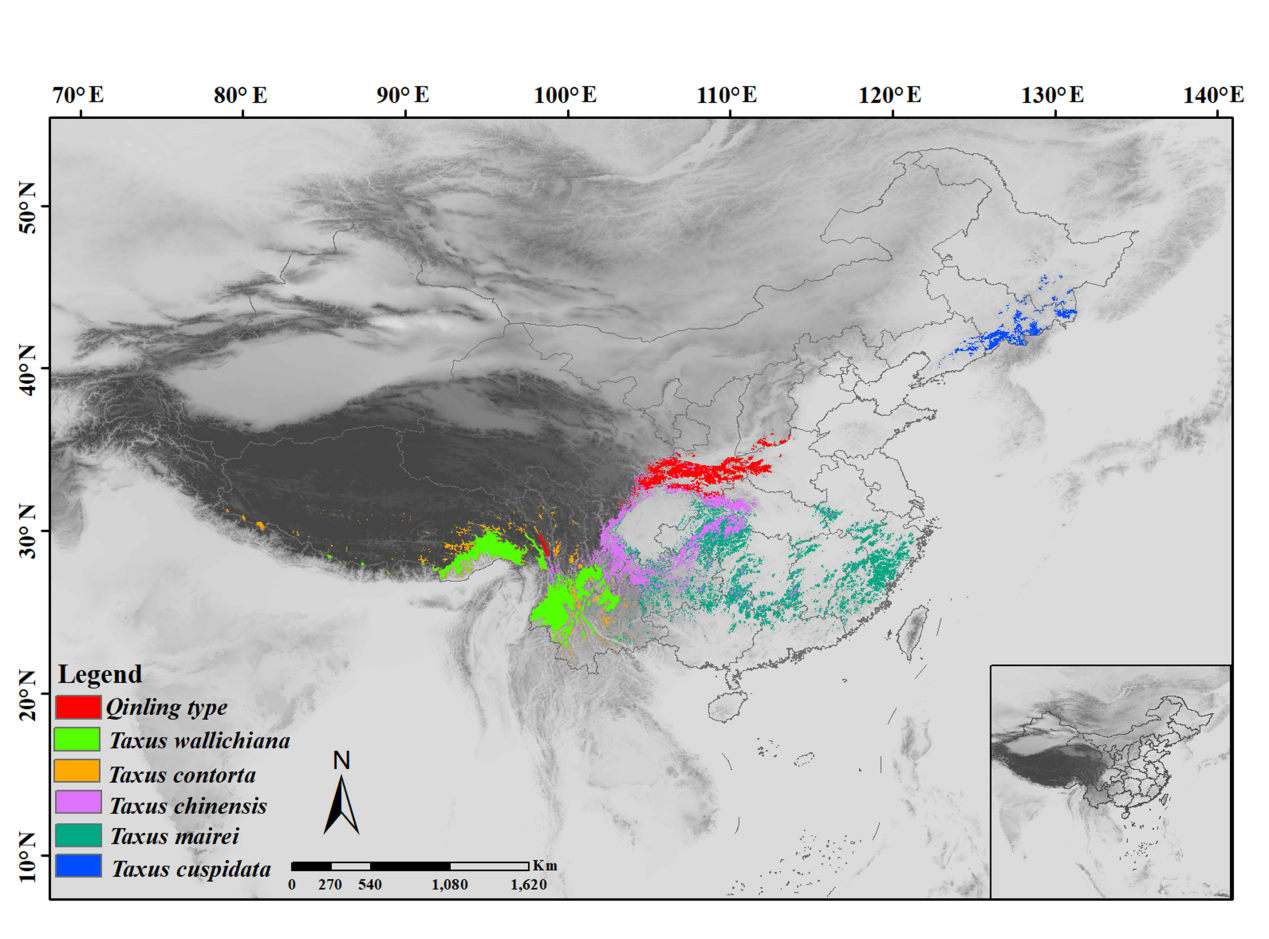
**

Fig. S2 Distribution map of *Taxus* species in China according to the Farjon (2017).


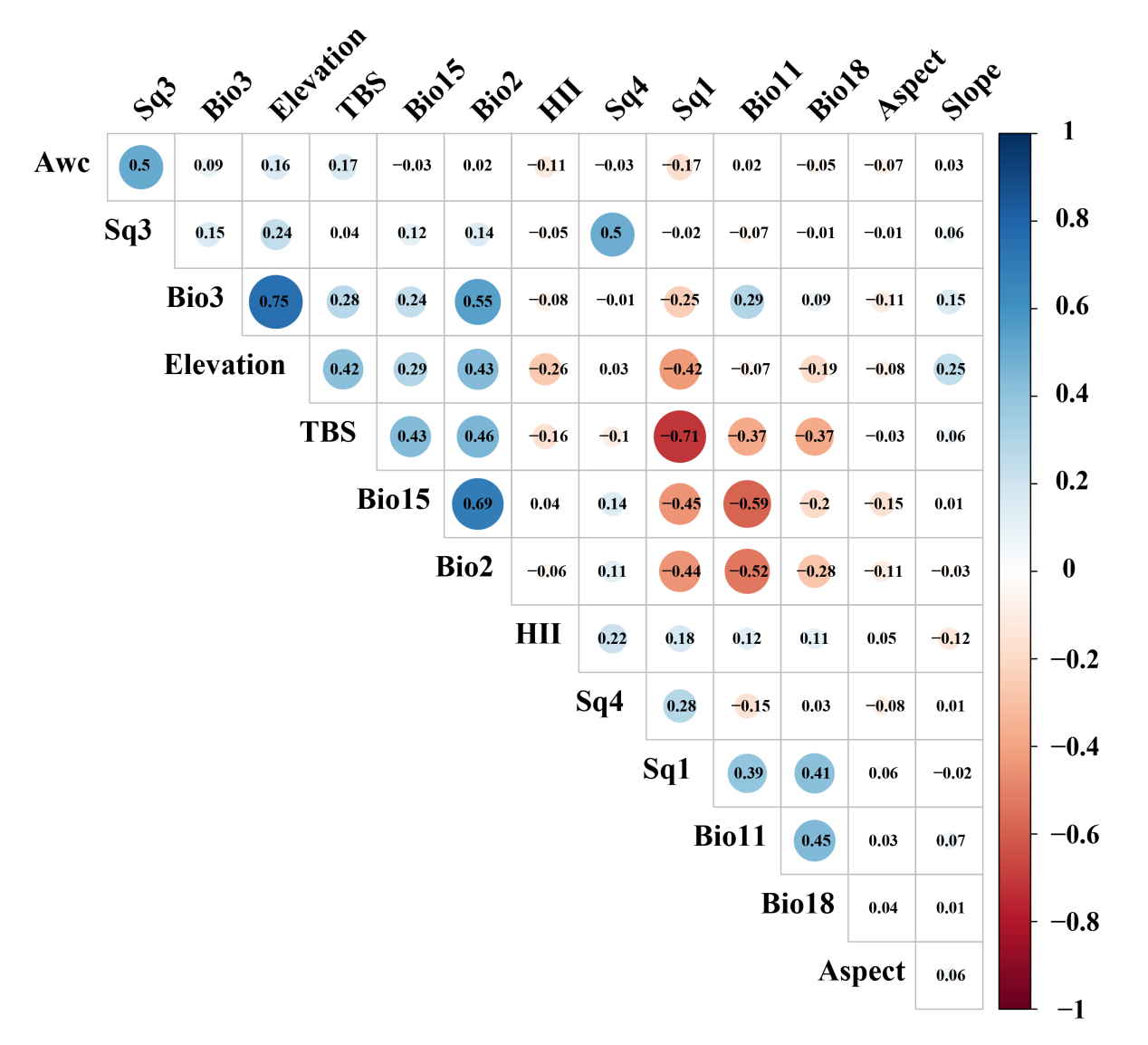


**Fig. S3** Correlation matrix of 14 bioclim variables for the six *Taxus* species in the niche overlap analysis.

Notes: Bio2: Mean Diurnal Range (Mean of monthly (max temperature - min temperature)); Bio3: Isothermality; Bio11: Mean Temperature of Coldest Quarter; Bio15: Precipitation Seasonality; Bio18: Precipitation of Warmest Quarter; Sq1: Nutrient availability; Sq3: Rooting conditions; Sq4: Oxygen availability to roots; TBS: Topsoil base saturation; AWC: Available water storage capacity; HII: Human influence index.

**Table S1** Multiple comparison of leaves between two species based on Kruskal-Wallis’s test.

| Comparison | p.adj | | |
| --- | --- | --- | --- |
|  | Length | Width | Length/Width |
| *Qinling type/T. wallichiana* | 5.84E-27 | 2.56E-32 | 7.75E-06 |
| *Qinling type/T. contorta* | 0^ns^ | 3.10E-45 | 2.11E-35 |
| *Qinling type/T. chinensis* | 2.64E-09 | 2.20E-02* | 3.35E-01^ns^ |
| *Qinling type/T. mairei* | 0^ns^ | 6.85E-06 | 2.88E-09 |
| *Qinling type/T. cuspidata* | 7.68E-05 | 7.17E-08 | 2.08E-12 |
| *T. wallichiana/T. contorta* | 3.36E-23 | 3.73E-01^ns^ | 4.05E-13 |
| *T. wallichiana/T. chinensis* | 5.83E-05 | 1.01E-17 | 4.15E-12 |
| *T. wallichiana/T. mairei* | 3.22E-29 | 2.07E-64 | 7.20E-29 |
| *T. wallichiana/T. cuspidata* | 2.10E-53 | 8.17E-09 | 2.62E-01^ns^ |
| 1. *contorta/T. chinensis* | 3.80E-07 | 1.46E-27 | 3.82E-49 |
| 1. *contorta/T. mairei* | 0^ns^ | 3.19E-82 | 2.56E-79 |
| 1. *contorta/T. cuspidata* | 1.18E-06 | 4.40E-16 | 2.48E-06 |
| 1. *chinensis/T. mairei* | 1.18E-10 | 2.92E-15 | 6.68E-04 |
| 1. *chinensis/T. cuspidata* | 1.10E-26 | 1.13E-01^ns^ | 5.37E-21*** |
| 1. *mairei/T. cuspidata* | 6.19E-04*** | 1.76E-26 | 6.18E-42 |

Notes:Those not marked in the upper right corner are all significant (****).

**Table S2** Sampling site of the *Qinling type.*

| Code | Location | Longititude/E | Latitude/N | Elevation/m | Number |
| --- | --- | --- | --- | --- | --- |
| ZS | Zhashui, Shaanxi | 109.393 | 33.575 | 933 | 2 |
| HB | Ankang, Shaanxi | 108.968 | 33.122 | 1237 | 6 |
| SC | Ningshan, Shaanxi | 108.438 | 33.338 | 1180 | 3 |
| YHS | Liuba, Shaanxi | 106.883 | 33.712 | 1250 | 3 |
| MB | Lueyang, Shaanxi | 105.778 | 33.349 | 1148 | 3 |
| SSW | Foping, Shaanxi | 107.999 | 33.566 | 910 | 2 |
| GP | Lantian, Shaanxi | 109.505 | 33.91 | 1388 | 2 |
| MAQ | Zhouzhi, Shaanxi | 108.034 | 33.911 | 1280 | 2 |
| ZZ | Zhouzhi, Shaanxi | 108.23 | 33.87 | 1100 | 6 |
| LW | Zhouzhi, Shaanxi | 108.06 | 33.83 | 1400 | 6 |
| MZP | Huyi, Shaanxi | 108.294 | 33.795 | 1380 | 2 |
| GYG | Fengxian, Shaanxi | 106.625 | 33.813 | 1260 | 3 |
| LMW | Taibai, Shaanxi | 107.121 | 33.941 | 1380 | 6 |
| TB | Meixian, Shaanxi | 107.904 | 34.133 | 659 | 2 |
| LD | Liangdang, Gansu | 106.34 | 33.81 | 1100 | 6 |
| HX | Huixian, Gansu | 106.34 | 33.73 | 950 | 4 |
| WN | Weinan, Shaanxi | 109.89 | 34.33 | 1500 | 6 |
| JDZ | Weinan, Shaanxi | 109.975 | 34.334 | 1515 | 2 |

**Table S3** Information of 102 sequences in current study.

| Source |  | Sample | ITS | *trn*L-*trn*F | *rbc*L | Voucher specimens |
| --- | --- | --- | --- | --- | --- | --- |
|  | Zhouzhi, Shaanxi | ZZ01 | MZ208838 | MZ220779 | MW893342 | WZZ201901 |
|  |  | ZZ04 | MZ208839 | MZ220780 | MW893343 | WZZ201904 |
|  |  | ZZ07 | MZ208840 | MZ220781 | MW893344 | WZZ201907 |
|  |  | ZZ11 | MZ208841 | MZ220782 | MW893345 | WZZ201911 |
|  |  | ZZ12 | MZ208842 | MZ220783 | MW893346 | WZZ201912 |
|  |  | ZZ15 | MZ208843 | MZ220784 | MW893347 | WZZ201915 |
|  | Weinan, Shaanxi | WN01 | MZ208844 | MZ220785 | MW893348 | WWN201901 |
|  |  | WN02 | MZ208845 | MZ220786 | MW893349 | WWN201902 |
|  |  | WN03 | MZ208846 | MZ220787 | MW893350 | WWN201903 |
| Current study |  | WN04 | MZ208847 | MZ220788 | MW893351 | WWN201904 |
|  |  | WN05 | MZ208848 | MZ220789 | MW893352 | WWN201905 |
|  |  | WN06 | MZ208849 | MZ220790 | MW893353 | WWN201906 |
|  |  | LW01 | MZ208850 | MZ220791 | MW893354 | WLW201901 |
|  |  | LW07 | MZ208851 | MZ220792 | MW893355 | WLW201907 |
|  | Zhouzhi, Shaanxi | LW11 | MZ208852 | MZ220793 | MW893356 | WLW201911 |
|  |  | LW14 | MZ208853 | MZ220794 | MW893357 | WLW201914 |
|  |  | LW19 | MZ208854 | MZ220795 | MW893358 | WLW201919 |
|  |  | LW21 | MZ208855 | MZ220796 | MW893359 | WLW201921 |
|  | Liangdang, Gansu | LD04 | MZ208856 | MZ220797 | MW893360 | WLD201904 |
|  |  | LD07 | MZ208857 | MZ220798 | MW893361 | WLD201907 |
|  |  | LD10 | MZ208858 | MZ220799 | MW893362 | WLD201910 |
|  |  | LD13 | MZ208859 | MZ220800 | MW893363 | WLD201913 |
|  |  | LD16 | MZ208860 | MZ220801 | MW893364 | WLD201916 |
|  |  | LD18 | MZ208861 | MZ220802 | MW893365 | WLD201918 |
|  | Huixian, Gansu | HX01 | MW788517 | MW792129 | MW792092 | WHX201901 |
|  |  | HX02 | MW788518 | MW792130 | MW792093 | WHX201902 |
|  |  | HX03 | MW788519 | MW792131 | MW792094 | WHX201903 |
|  |  | HX04 | MW788520 | MW792132 | MW792095 | WHX201904 |
|  | Huyi, Shaanxi | MZP1 | OQ891381 | OQ978853 | OQ913085 | LMZP20211 |
|  |  | MZP2 | OQ891382 | OQ978854 | OQ913086 | LMZP20212 |
|  | Zhouzhi, Shaanxi | MAQ1 | OQ891385 | OQ978857 | OQ913089 | LMAQ20211 |
|  |  | MAQ2 | OQ891386 | OQ978858 | OQ913090 | LMAQ20212 |
| Current study | Ningshan, Shaanxi | SC1 | OQ891387 | OQ978859 | OQ913091 | LSC20211 |
|  |  | SC2 | OQ891388 | OQ978860 | OQ913092 | LSC20212 |
|  |  | SC4 | OQ891389 | OQ978861 | OQ913093 | LSC20214 |
|  | Lantian, Shaanxi | GP1 | OQ891391 | OQ978863 | OQ913095 | LGP20211 |
|  |  | GP2 | OQ891392 | OQ978864 | OQ913096 | LGP20212 |
|  | Weinan, Shaanxi | JDZ1 | OQ891400 | OQ978872 | OQ913104 | LJDZ20211 |
|  |  | JDZ2 | OQ891401 | OQ978873 | OQ913105 | LJDZ20212 |
|  | Lueyang, Shaanxi | MB1 | OQ891402 | OQ978874 | OQ913106 | LMB20211 |
|  |  | MB2 | OQ891403 | OQ978875 | OQ913107 | LMB20212 |
|  |  | MB3 | OQ891404 | OQ978876 | OQ913108 | LMB20213 |
|  | Taibai, Shaanxi | LWM1 | OQ891408 | OQ978880 | OQ913112 | LLMW20211 |
|  |  | LWM2 | OQ891409 | OQ978881 | OQ913113 | LLMW20212 |
|  |  | LWM3 | OQ891410 | OQ978882 | OQ913114 | LLMW20213 |
|  |  | LWM4 | OQ891411 | OQ978883 | OQ913115 | LLMW20214 |
|  |  | LWM5 | OQ891412 | OQ978884 | OQ913116 | LLMW20215 |
|  |  | LWM6 | OQ891413 | OQ978885 | OQ913117 | LLMW20216 |
|  | Meixian, Shaanxi | TB1 | OQ891424 | OQ978896 | OQ913128 | LTB20211 |
|  |  | TB2 | OQ891425 | OQ978897 | OQ913129 | LTB20212 |
|  | Fengxian, Shaanxi | GYG1 | OQ891429 | OQ978901 | OQ913133 | LGYG20211 |
|  |  | GYG2 | OQ891430 | OQ978902 | OQ913134 | LGYG20212 |
| Current study |  | GYG3 | OQ891431 | OQ978903 | OQ913135 | LGYG20213 |
|  | Zhashui, Shaanxi | ZS1 | OQ891432 | OQ978904 | OQ913136 | LZS20211 |
|  |  | ZS2 | OQ891433 | OQ978905 | OQ913137 | LZS20212 |
|  | Ankang, Shaanxi | HB1 | OQ891434 | OQ978906 | OQ913138 | LHB20211 |
|  |  | HB2 | OQ891435 | OQ978907 | OQ913139 | LHB20212 |
|  |  | HB3 | OQ891436 | OQ978908 | OQ913140 | LHB20213 |
|  |  | HB4 | OQ891437 | OQ978909 | OQ913141 | LHB20214 |
|  |  | HB5 | OQ891438 | OQ978910 | OQ913142 | LHB20215 |
|  |  | HB6 | OQ891439 | OQ978911 | OQ913143 | LHB20216 |
|  | Liuba, Shaanxi | YHS1 | OQ891442 | OQ978914 | OQ913146 | LYHS20211 |
|  |  | YHS2 | OQ891443 | OQ978915 | OQ913147 | LYHS20212 |
|  |  | YHS4 | OQ891444 | OQ978916 | OQ913148 | LYHS20214 |
|  | Foping, Shaanxi | SSW1 | OQ891398 | OQ978870 | OQ913102 | LSSW20211 |
|  |  | SSW2 | OQ891399 | OQ978871 | OQ913103 | LSSW20212 |
|  | Taibai, Shaanxi | *Qinling type1* | HM590961 | HM591141 | HM591048 | MMO 05-694 |
|  | Lingchuan, Shanxi | *Qinling type2* | HM590963 | HM591143 | HM591050 | LJ-05-1062 |
|  | Lushi, Henan | *Qinling type3* | HM590962 | HM591142 | HM591049 | LJ-05-1024 |
| NCBI | Ningqiang, Shaanxi | *Taxus chinensis4* | MH267472 | HM591128 | HM591035 | MMO 05-597 |
|  | Chenggu, Shaanxi | *Taxus chinensis5* | HM590948 | HM591129 | HM591036 | MMO 05-507 |
|  | Shennongjia, Hubei | *Taxus chinensis6* | HM590949 | MH267568 | MH267389 | Liuj-TC-1 |
|  | Nanjiang, Sichuan | *Taxus chinensis7* | MH267473 | MH267569 | MH267390 | Liuj-TC-2 |
|  | Pingwu, Sichuan | *Taxus chinensis8* | HM590947 | HM591127 | HM591034 | LJ-05-999 |
|  | Houzhenzi,Shaanxi | *Taxus chinensis9* | MZ208893 | MZ220834 | MW893397 | WHZZ09 |
|  | Houzhenzi,Shaanxi | *Taxus chinensis10* | MZ208894 | MZ220835 | MW893398 | WHZZ10 |
|  | Langao,shaanxi | *Taxus chinensis11* | MZ208899 | MZ220840 | MW893403 | WLGM11 |
|  | Langao,shaanxi | *Taxus chinensis12* | MZ208900 | MZ220841 | MW893404 | WLGM12 |
|  | Linan, Zhejiang | *Taxus mairei13* | HM590960 | HM591140 | HM591047 | ZHXM050691 |
|  | Fuqing, Fujian | *Taxus mairei14* | HM590959 | HM591139 | HM591046 | ZHXM0506152 |
|  | Xishuanbanna, Yunnan | *Taxus mairei15* | MH267424 | HM591138 | MH267354 | 20150706-02 |
| NCBI | Jinxiu, Guangxi | *Taxus mairei16* | HM590958 | HM591138 | HM591045 | GLM-05971 |
|  | Lichuan, Hubei | *Taxus mairei17* | HM590957 | HM591137 | HM591044 | Zeng7-10 |
|  | Weixin, Sichuan | *Taxus mairei18* | HM590956 | HM591136 | HM591043 | GLM-07623 |
|  | Tengchong, Yunnan | *Taxus mairei19* | MH267488 | MH267584 | MH267405 | LJ-06091 |
|  | Kunming, Yunnan | *Taxus mairei20* | MH267433 | HM591138 | MH267363 | KZH01 |
|  | Qujing, Yunnan | *Taxus mairei21* | MH267444 | HM591139 | MH267374 | 154252 |
|  | Dali, Yunan | *Taxus mairei22* | MH267447 | HM591138 | MH267377 | SYJT2A |
|  | Cuona, Xizang | *Taxus wallichiana23* | HM590941 | HM591121 | HM591028 | GLM-081828 |
|  | Chayu, Xizang | *Taxus wallichiana24* | HM590936 | HM591116 | HM591022 | GLM-2149 |
|  | Gongshan,Yunnan | *Taxus wallichiana25* | EF680253 | EF680273 | HM591026 | GLM-2301 |
|  | Jingdong, Yunnan | *Taxus wallichiana26* | HM590938 | HM591118 | HM591024 | GM-24222 |
|  | Luquan, Yunnan | *Taxus wallichiana27* | HM590939 | HM591119 | HM591025 | LJ-09228 |
|  | Jingping, Yunnan | *Taxus wallichiana28* | HM590937 | HM591117 | HM591023 | GLM-06257 |
|  | Solukhumbu District,Nepal | *Taxus wallichiana29* | HM590942 | HM591122 | HM591029 | NEP001-0364 |
|  | Yunlong, Yunnan | *Taxus wallichiana30* | MH267461 | JQ406945 | MH267381 | KZH06 |
|  | Linzhi, Xizang | *Taxus wallichiana31* | HM590940 | HM591120 | HM591027 | LJ-07035 |
|  | Linjiang, Jilin | *Taxus cuspidata32* | HM590969 | HM591147 | HM591056 | LJM-48 |
|  | Shiraki-mine, Honshu | *Taxus cuspidata33* | HM590971 | HM591149 | HM591058 | Kokubugata-6972 |
| NCBI | Chamoli, Gopeshwar | *Taxus contorta34* | HM590974 | HM591152 | HM591062 | Yuhy-74 |
|  | Jilong , Xizang | *Taxus contorta35* | HM590975 | HM591153 | HM591063 | GLM-081628 |
|  | Changsha,Hunan | *Pseudotaxus chienii* | MW788529 | MW792141 | MW792104 | WZWY201901 |

**Notes:** Voucher samples obtained from NCBI are sourced from Liu et al. (2018)[29] and Wu (2021)[42].

**Table S4** Information of DNA barcoding.

| Primer | 5'-3' | Amplification procedures | Source |
| --- | --- | --- | --- |
| *rbc*L | 1F:ATGTCACCACAAACAGAAAC | 94℃ 4min,  (94℃ 45s, 54℃ 1min, 72℃ 1min)×35cycles,  72℃ 7min | Fay et al., 1997 |
|  | 724R:TCGCATGTACCTGCAGTAGC |  |  |
| *trn*L-*trn*F | trnc:CGAAATCGGTAGACGCTACG | 95℃ 2min,  (94℃ 1min, 55℃ 30s, 72℃ 1min)×34cycles,  72℃ 7min | Taberlet et al., 1991 |
|  | trnf:ATTTGAACTGGTGACACGAG |  |  |
| ITS | 18sF:GCGGTAGGATCATTGTCG | 94℃ 3min,  (94℃ 30s, 60℃ 30s, 72℃ 1min30s)×25cycles,  72℃ 7min | Yin et al., 2020 |
|  | 18sR:TCCTCCGCTTATTGATATGC |  |  |
